# Determining whether LV filling time contributes to HF symptoms in different widths of QRS in LBBB patients: A clinical study

**DOI:** 10.21542/gcsp.2024.8

**Published:** 2024-01-03

**Authors:** Masoumeh Ahmadzadeh, Mehran Rahimi, Mehrnoush Toufan-Tabrizi*, Kamran Mohammadi

**Affiliations:** 1 Cardiovascular Research Center, Tabriz University of Medical Sciences, Tabriz, Iran

## Abstract

Objectives: Accurate assessment of left ventricular (LV) function is essential for managing patients with left bundle branch block (LBBB). This study aimed to evaluate the relationship between LV systolic function, left ventricular diastolic filling time (LVFT), QRS duration, and heart failure symptoms in patients with LBBB.

Methods: This study was conducted between June 2021 and June 2022. Patients with LBBB and sinus rhythm who were referred to the echocardiography department were included in the study. All the patients underwent electrocardiogram-gated echocardiography using the same machine. In this study, the LVFT value was measured in absolute terms and as a ratio to the R-R interval (LVFT/RR).

Results: A total of sixty-five patients were included, forty-two (64.6%) were women, and the mean age was 60.71 ± 8.72. We performed three one-way ANOVA tests that showed that LV filling time/RR ratio, QRS duration, and ejection fraction were significantly different between heart failure classes (*p* = 0.008, *p* = 0.001, and *p* < 0.001, respectively). A weak correlation was observed between LVEF and LVFT/RR (*r* = 0.349, *p* = 0.004). Additionally, QRS duration was negatively correlated with LVEF (*r* =  − 0.395, *p* = 0.004) and LVFT/RR (*r* =  − 0.350, *p* = 0.004), although these correlations were weak.

Conclusion: We showed that LVFT/RR ratio differed significantly between HF functional classes and was lower in patients with more severe HF symptoms. Additionally, QRS duration was negatively correlated with LVEF and LVFT/RR, and patients with more severe HF symptoms had longer QRS durations.

## Introduction

Left bundle branch block (LBBB) has an estimated prevalence of 0.2%−1.1% in the general population, which increases with age. It rises from less than 1% in the 50s to 6% in the 80s. Compared with patients with normal conduction or right bundle branch block (RBBB), LBBB patients have lower left ventricular (LV) systolic function, worse prognosis, and higher rates of cardiovascular mortality, sudden cardiac death, coronary artery disease, and heart failure (HF)^1,2^.

An accurate assessment of LV function is essential for the management of patients with LBBB^3^. Echocardiography is a widely used non-invasive imaging modality for evaluating LV function. Previous studies using 2D echocardiography have shown a decrease in LV ejection fraction (LVEF) and an increase in LV volume in patients with LBBB^4,5^. However, LVEF may not always indicate the presence or severity of heart failure symptoms^6^.

LBBB can lead to a delay in the contraction of the left ventricle, which can affect the onset of diastole and ventricular filling. Additionally, uncoordinated ventricular contractions in LBBB may lead to left ventricular contractile inefficiency^7^. LV filling time (LVFT) refers to the time required for the left ventricle to fill during diastole. Thus, LVFT measures the time interval between the mitral valve opening and closure, which reflects the duration of diastolic filling of the left ventricle. LVFT is an important determinant of cardiac output^8^. Previous studies have shown that LVFT may be reduced in patients with LBBB through the prolongation of functional mitral regurgitation, which can lead to impaired cardiac output and heart failure symptoms^7,9,10^.

Nevertheless, there is little information regarding the relationship between LV filling time and QRS width in patients with LBBB. Prolonged QRS duration (>0.10 s) is a specific indicator of decreased LV systolic function^11^. However, the relationship between the width of the QRS complex and LVFT in LBBB patients with signs of heart failure is not well understood. The principal investigator’s hypothesis based on her clinical practice was that the presence of left bundle branch block in patients with similar QRS width can result in different LVEF and heart failure symptoms, indicating that the left ventricular filling time during diastole could explain this variability. Additionally, signs of heart failure do not always follow the level of ejection fraction^12^. Thus, the purpose of this study was to determine the relationship between the width of the QRS complex and left ventricular filling time as well as to determine the relationship with signs of heart failure in these patients.

## Methods

This study was conducted between June 2021 and June 2022 at Shahid Madani Heart Center of Tabriz University of Medical Sciences. The medical ethics committee of Tabriz University of Medical Sciences approved this study. All patients with LBBB and sinus rhythm referred to the echocardiography department were included. Patients with a pacemaker, heart rhythm other than sinus rhythm, premature atrial contractions (PAC) or premature ventricular contractions (PVC), transient atrial fibrillation, intermittent LBBB, severe valvular disease, poor quality of acoustic window, and any lack of patient consent to continue the study were excluded. Written informed consent was obtained from all patients.

The 12-lead surface electrocardiogram was recorded at 25 mm/s at rest (Medical ECONET, CARDIO M 12) immediately before echocardiography. QRS duration, and RR interval were measured. The R-R cycle is defined as the time interval between two successive R waves measured in milliseconds. LBBB was detected according to diagnostic criteria defined by the American College of Cardiology (ACC) and American Heart Association (AHA): QRS duration (the time between the onset of Q-wave and end of S-wave) greater than 120 ms, presence of either a QS or a small r wave with a large S wave in Lead V1, a notched R wave in Lead V6 without Q wave^13^. Complete medical history and relevant history of heart failure were obtained. The New York Heart Association (NYHA) functional classification was then determined for each patient. The investigators who took the medical history were blinded to echocardiography and ECG data.

### Echocardiography

A complete transthoracic echocardiography study using a commercially available ultrasound machine (Philips Affinity 70 device, USA) was performed and interpreted in accordance with American Society of Echocardiography guideline^14^. LVEF was measured using the biplane Simpson method. Pulsed-wave Doppler was used to record mitral inflow for three to five cardiac cycles at the level of the mitral valve  leaflet tips. The time interval between the onset of mitral valve inflow (E-wave) and the end of atrial contraction (A-wave) on the spectral Doppler waveform was measured as diastolic LV filling time in four consecutive cardiac cycles (Figure 1A)^15^. In this study, the LV filling time was measured in absolute terms and its ratio to the R-R interval (LVFT/RR) (Figure 1B). Both values were measured simultaneously during ECG-gated echocardiography. The R-R cycles and filling times were measured simultaneously over four consecutive cycles. All images were stored digitally and reviewed offline.

**Figure 1a.**
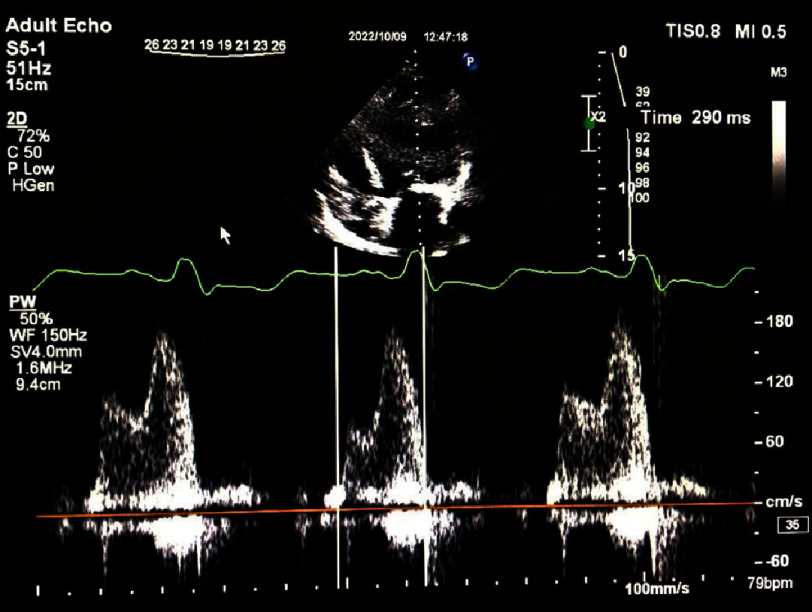
An apical four-chamber view (pulsed doppler recording) showing LV filling time =290 ms.

**Figure 1b.**
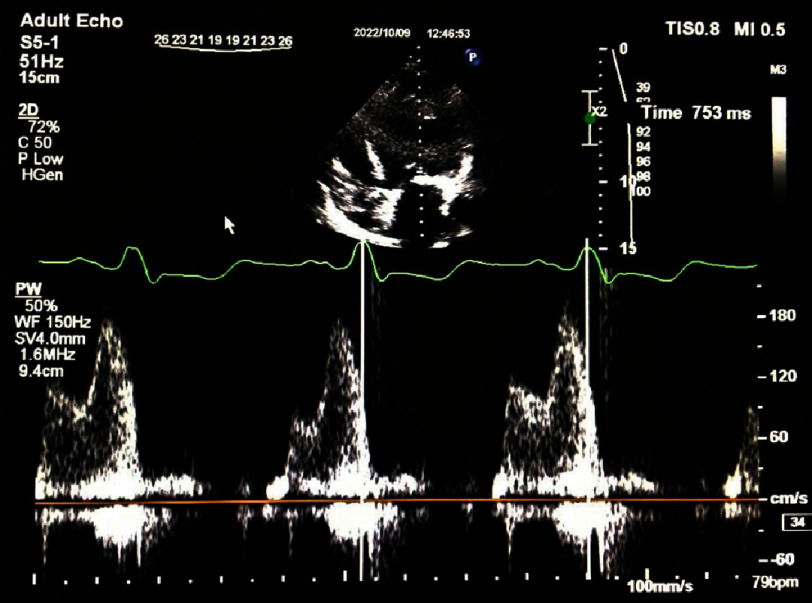
R-R interval duration.

### Statistical analysis

The results of descriptive statistics were expressed as frequency and percentage for qualitative variables and as mean ±  standard deviation for normal quantitative variables. The normal distribution of the data was checked using the Kolmogorov–Smirnov analysis. Pearson correlation was used to determine the relationship between two quantitative variables, and a chi-square test was used to determine the relationship between two nominal qualitative variables. Using the independent *t*-test (*t*-test), we determined a quantitative variable’s mean among the levels of a two-way qualitative variable. We calculated a quantitative variable’s mean among the levels of a three-way qualitative variable using one-way ANOVA and Tukey’s post hoc analysis. SPSS version 26 statistical software was used to perform all analyses. *P* value < 0.05 was considered significant in all tests.

## Results

A total of 138 patients were included in this study. Seventy-three patients experienced frequent premature ventricular contractions (PVC) or frequent PACs/transient atrial fibrillation during the echocardiography study, which precludes the exact definition of LVEF, RR interval, and LV filling time. Finally, we included sixty-five patients who met the inclusion criteria were included. The baseline characteristics of the patients and the electrocardiographic and echocardiographic data are shown in Tables 1 and 2, respectively. Five patients exhibited myocardial wall thinning, with two showing thinning in the LAD artery territory.

**Table 1:** Baseline clinical and echocardiographic data.

Age, years		60.71 ± 8.723
Female sex, *n* (%)		42 (64.6)
Hypertension, *n* (%)		47 (72.3)
Heart Failure, *n* (%)		28 (43.08)
Referred for, *n* (%)	DHF	17 (26.2)
	ACS	25 (38.5)
	Syncope	1 (1.5)
	CVA	1 (1.5)

**Notes.**

Data are presented as mean ± SD or number (frequency).

ACS Acute Coronary Syndrome DHF Decompensated Heart failure

**Table 2:** Electrocardiographic and echocardiographic data.

QRS width, ms		145.28 ± 13.933
RR interval, ms		808.08 ± 134.70
ECG axis, *n* (%)	Normal axis	29 (44.62%)
	Left-axis deviation	36 (55.39%)
	Right-axis deviation	0 (0%)
LVEF, %		38.5 ± 12.6
LV filling time, ms		359.66 ± 102.885
LV filling time / RR		0.431 ± 0.073
SPWMD, ms		173.38 ± 26.597

**Notes.**

Data are presented as mean ± SD or number (frequency).

SPWMD Septal to Posterior Wall Motion Delay

To investigate the relationship between LV filling time and RR interval with heart failure (HF) functional classes, a one-way ANOVA was performed. The results showed that the LVFT/RR ratio differed significantly between HF functional classes (*p* = 0.008). This indicates that the LVFT/RR ratio was significantly lower in patients with more severe HF symptoms. Subsequent Tukey post hoc analysis revealed that this significant difference was mainly due to the difference between individuals in NYHA class III and those without HF (Mean Difference: 0.06, *p* = 0.006) (Table 3). However, the absolute value of LV filling time did not differ significantly between patients in different NYHA functional classes. Table 4 shows the values of LVEF, absolute LVFT, LVFT/RR, and HF symptom presence based on the three QRS duration categories. The analysis revealed that LVEF, absolute LVFT, and LVFT/RR were lower in patients with longer QRS duration. However, the analysis was only significant for the differences in LVEF between QRS duration categories. Additionally, patients with longer QRS durations were more likely to experience heart failure symptoms.

**Table 3:** One way ANOVA results of assessing the difference in LVFT/RR among NYHA classes.

**ANOVA**								
**ECHO- LV filling time / RR**								
		**N**	**Mean**	**Std. Deviation**	**95% Confidence Interval for Mean**		**F**	**p-value**
					**Lower Bound**	**Upper Bound**		
**HF. positive**	**None**	37	0.454	0.057	0.435	0.473	5.222	0.008
	**Stage II**	6	0.425	0.090	0.329	0.520		
	**Stage III**	22	0.394	0.080	0.358	0.430		

**Table 4:** Echocardiographic results based on QRS categories.

QRS duration	120-139 (*n* = 23)	140-159 (*n* = 36)	160-179 (*n* = 6)	*P* value
Parameter				
LVEF, %	46.30 ± 8.82	33.97 ± 12.56	36.17 ± 13.36	0.001
LVFT, ms	363.48 ± 82.26	362.61 ± 108.12	327.33 ± 150.0	0.727
LVFT/RR	45.09 ± 5.41	42.70 ± 7.70	38.5 ± 10.30	0.127
No HF symptoms, *n* (%)	21 (91.3%)	14 (38.89%)	2 (33.33%)	<0.001
HF symptoms present, *n* (%)	2 (8.7%)	22 (61.11%)	4 (66.67%)	

**Notes.**

Data are presented as mean ±  SD or number (frequency).

LVEF Left ventricular ejection fraction LVFT Left ventricular filling time LVFT/RR Left ventricular filling time to R-R interval HF Heart failure

**Figure 2.**
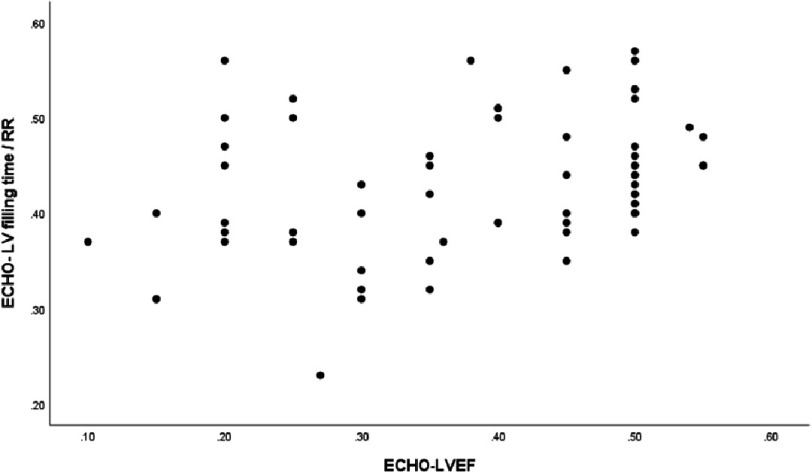
The plot shows the relationship between left ventricle filling time/RR and left ventricle ejection fraction (*r* = 0.349, *P*-value =0.004).

**Figure 3.**
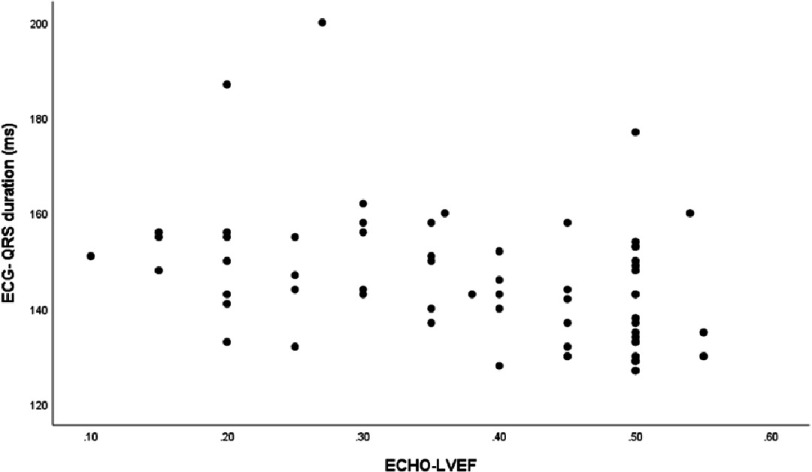
The plot shows the relationship between QRS duration and Left ventricle ejection fraction (r =−0.395, *P*-value =0.004).

**Figure 4.**
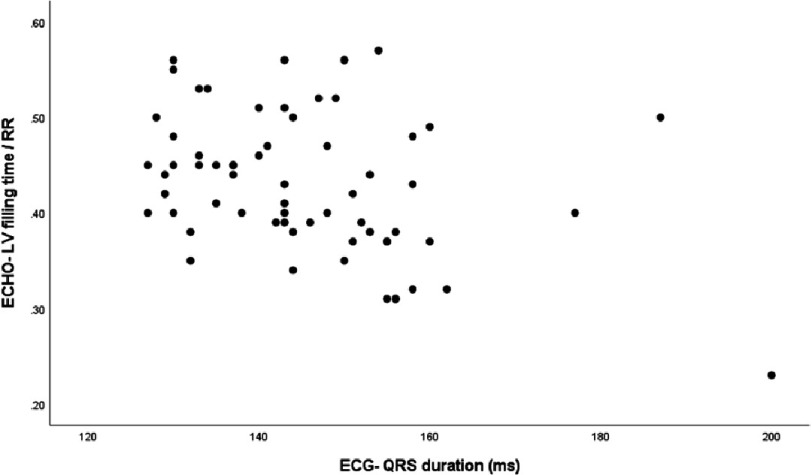
The plot shows the relationship between left ventricle filling time/RR and QRS duration (*r*=−0.350, *P*-value =0.004).

There was a weak correlation between LVEF and LVFT/RR (*r* = 0.349, *p* = 0.004) (Figure 2). Additionally, QRS duration was negatively correlated with LVEF (r = −0.395, *p* = 0.004) (Figure 3) and LVFT/RR (*r* = −0.350, *p* = 0.004) (Figure 4), although these correlations were weak. However, there was no significant correlation between absolute LVFT and QRS duration (*r* =−0.111, *p* = 0.380) or between absolute LVFT and LVEF (*r* = 0.181, *p* = 0.150).

Another one-way ANOVA test was conducted to examine the differences in QRS duration between the different classes of HF. The results showed a significant difference in QRS duration between HF classes (*p* = 0001), and a subsequent Tukey post hoc analysis revealed that this statistically significant difference was due to the difference in QRS duration between patients with NYHA class III and those without HF. Thus, patients with more severe HF symptoms had longer QRS duration. In addition, the ejection fraction differed significantly between classes of heart failure (*p* < 0.001), with LVEF showing significant differences between individuals without heart failure and all classes of heart failure.

## Discussion

In this prospective study, patients with LBBB and normal sinus rhythm with different NYHA functional classes of heart failure were investigated. We evaluated the relationship between left ventricular filling time (LVFT), LVFT/RR ratio, and QRS duration as well as their association with heart failure symptoms and left ventricular ejection fraction (LVEF). The results of this study demonstrate that patients with left bundle branch block (LBBB), sinus rhythm, and more severe heart failure symptoms exhibit a wider QRS width, lower LVFT/RR ratios, and lower ejection fractions. Our findings suggest that QRS duration is negatively correlated with LVEF in patients with LBBB. QRS duration also appears to be a reflection of LV ejection fraction, in line with the study by Kim and colleagues^16^. A study of 23 patients with normal conduction and 12 patients with complete LBBB reported that patients with complete LBBB had higher end-systolic and diastolic volumes and a lower EF than patients with normal conduction^17^. Furthermore, previous studies have found that QRS duration is related to inter- and intraventricular dyssynchrony and a type of abnormal conduction-induced cardiomyopathy, as recently reported by Jose Huizar and colleagues^18–20^.

Patients with LBBB have a prolonged LV ejection time, which reduces the diastolic filling time^21^. Chronically reduced diastolic filling time may cause chronic LV underfilling, increased left atrial mean pressure and symptomatic HF with preserved EF, which is more prominent at faster heart rates. It may also be a marker of LV dysfunction severity owing to LBBB-induced dyssynchrony^22^. LVFT divided by RR interval duration (LVFT/RR) is a parameter representing atrioventricular dyssynchrony and is linked to left ventricular reverse remodeling^23^. Patients with cardiomyopathy exhibit higher LVFT/RR values but lower LVFT values than those with ischemic heart disease^24^. Regarding the clinical implications of LVFT, the PROSPECT study showed that pre-implantation LVFT ≤ 40% is significantly associated with 6-month improvements in heart failure symptoms and quality of life with cardiac resynchronization therapy (CRT). A sub-analysis of the PROSPECT study revealed that patients who experienced a 15% or greater reduction in LV end-systolic volume and improved clinical composite score in response to CRT had lower LVFT/RR^23^. Our study found that patients with more severe HF symptoms had a lower LVFT/RR.

In line with other studies, LVFT/RR was weakly correlated with LV ejection fraction, and there was a weak negative correlation between LVFT/RR and QRS duration^25^. Notably, there was no significant correlation between absolute LVFT and QRS duration or between absolute LVFT and LVEF. Vancheri et al. did not find a significant correlation between the QRS duration and LVFT^26^. However, Charisopoulou and colleagues found that LVFT was positively correlated with stroke volume^27^. These results indicate that LVFT/RR is a much better choice for echocardiography evaluation because of its correlation with QRS duration and LVEF.

We found that QRS duration negatively correlated with LVEF in patients with LBBB. According to the one-way ANOVA analysis, QRS durations were significantly longer in patients with higher NYHA classes. Studies with a large study population have confirmed that the duration of QRS is significantly wider in NYHA classes III-IV than in NYHA class II^28,29^. However, NYHA class is not independently associated with QRS prolongation^29^. Multiple studies have demonstrated that QRS duration predicts mortality in patients with heart failure^18,30^. There are conflicting reports about the role of QRS duration in the long-term survival of heart failure patients. In a cohort of 973 patients with heart failure, the association between QRS duration and long-term survival was not statistically significant^31^. However, after one year of follow-up in 5517 patients of the Italian Network on CHF Registry, the authors concluded that in patients with CHF, LBBB was an unfavorable prognostic marker^32^. Unfortunately, we could not follow patients for MACE.

## Limitations

This study had some limitations. First, we lost 73 patients due to frequent PVC or transient AF, which reduced the study population. Second, we did not assess the role of different medications taken by patients. However, this issue was solved by employing normalized LV filling time (LVFT/RR). This ratio counterbalances the effect of *β*-blockers and other rate-controlling drugs on the LV filling time.

## Conclusion

In this study, we showed that LVFT/RR ratio differed significantly between HF functional classes and was lower in patients with more severe HF symptoms. Additionally, QRS duration was negatively correlated with LVEF and LVFT/RR, and patients with more severe HF symptoms had longer QRS durations.

## Declarations

## Ethics approval and consent to participate

This article is part of a fellowship research project on echocardiography approved by the Ethics Committee at Tabriz University of Medical Sciences (IR.TBZMED.REC.1401.348). All patients provided written informed consent to participate in the study.

## Availability of data and materials

The datasets used and analyzed during the current study are available from the corresponding author upon reasonable request.

## Competing interests

The authors declare that they have no conflicts of interest.

## Funding

Tabriz University of Medical Sciences, Tabriz, Iran.

## Authors’ contributions

Conceptualization: Mehrnoush Toufan-Tabrizi. Data curation: Masoumeh Ahmadzadeh, Mehran Rahimi. Software: Mehran Rahimi. Methodology: Mehrnoush Toufan-Tabrizi, Kamran Mohammadi, Masoumeh Ahmadzadeh. Writing - original draft: Mehran Rahimi Masoumeh Ahmadzadeh. Writing - review & editing: Mehrnoush Toufan-Tabrizi, Kamran Mohammadi. Supervision: Mehrnoush Toufan-Tabrizi

## Acknowledgement

The authors acknowledge and thank Dr. Morteza Ghojazadeh, Ph.D., for his important contributions to this effort.
